# Alcohol harm reduction advertisements: a content analysis of topic, objective, emotional tone, execution and target audience

**DOI:** 10.1186/s12889-017-4218-7

**Published:** 2017-04-11

**Authors:** Kimberley Dunstone, Emily Brennan, Michael D. Slater, Helen G. Dixon, Sarah J. Durkin, Simone Pettigrew, Melanie A. Wakefield

**Affiliations:** 1 Centre for Behavioural Research in Cancer, Cancer Council Victoria, 615 St Kilda Road, Melbourne, VIC 3004 Australia; 2 Ohio State University, 3022 Derby Hall, 154 North Oval Mall, Columbus, OH 43210 USA; 3 School of Psychology and Speech Pathology, Curtin University, Kent Street, Bentley, WA 6102 Australia

**Keywords:** Alcohol, Alcohol harm reduction, Mass media campaigns, Population health, Content analysis, Advertising characteristics

## Abstract

**Background:**

Public health mass media campaigns may contribute to reducing the health and social burden attributed to alcohol consumption, but little is known about which advertising characteristics have been used, or have been effective, in alcohol harm reduction campaigns to date. As a first step towards encouraging further research to identify the impact of various advertising characteristics, this study aimed to systematically identify and examine the content of alcohol harm reduction advertisements (ads).

**Method:**

Ads were identified through an exhaustive internet search of Google, YouTube, Vimeo, and relevant government and health agency websites. Eligible ads were: English language, produced between 2006 and 2014, not primarily focused on drink-driving or alcohol in pregnancy, and not alcohol industry funded. Systematic content analysis of all ads was performed; each ad was double-coded.

**Results:**

In total, 110 individual ads from 72 different alcohol harm reduction campaigns were identified, with the main source countries being Australia (40%) and the United Kingdom (26%). The dominant topic for 52% of ads was short-term harms, while 10% addressed long-term harms, 18% addressed underage drinking, 17% communicated a how-to-change message, and 3% advocated for policy change. The behavioural objective of most ads was to motivate audiences to reduce their alcohol consumption (38%) or to behave responsibly and/or not get drunk when drinking (33%). Only 10% of all ads mentioned low-risk drinking guidelines. Eighty-seven percent of ads used a dramatisation execution style and 74% had a negative emotional tone. Ninety percent of ads contained messages or content that appeared to target adults, and 36% specifically targeted young adults.

**Conclusions:**

Some message attributes have been employed more frequently than others, suggesting several promising avenues for future audience or population-based research to compare the relative effectiveness of different characteristics of alcohol harm reduction ads. Given most alcohol-attributable harm is due to long-term disease, these findings suggest future campaigns may fill a potentially important gap if they were to focus on long-term harms. There is scope for such long-term harm campaigns to place greater emphasis on encouraging reduced personal consumption of alcohol, potentially through more frequent communication of low-risk drinking guidelines.

**Electronic supplementary material:**

The online version of this article (doi:10.1186/s12889-017-4218-7) contains supplementary material, which is available to authorized users.

## Background

Alcohol consumption is common, particularly in western societies [1], and a substantial proportion of drinkers consume alcohol at levels that increase their risk of immediate as well as long-term harm [2–4]. Alcohol use ranks among the top three risk factors for global disease burden [5], leading to about 3.3 million deaths annually [1]. A key impediment to efforts to reduce alcohol-related harm is the pervasive marketing of alcohol; estimates of annual industry advertising expenditure range from $220 million in Australia [6] to £200 million in the United Kingdom [7] and $3.5 billion in the United States [8]. These investments are made across traditional media such as television, print, radio, and billboards, in addition to internet advertising, social media, sponsorships, in-store promotions, and product placements [6, 9]. A substantial proportion is spent on television to advertise and promote alcohol products [6–8]. This marketing stimulates consumption by reinforcing alcohol-related norms and creating positive expectations about drinking experiences [8, 10, 11]. As a result of the industry’s considerable investment in alcohol marketing, drinking decisions are made in the context of a vast information asymmetry that emphasises the benefits of alcohol consumption and minimises information about potential harms.

Public health mass media campaigns are one way to redress this information imbalance and potentially reduce alcohol-related harm. In health promotion, mass media campaigns have been shown to be effective in changing health behaviours when implemented as part of a comprehensive approach that includes evidence-informed messages and a consistently high level of investment to ensure widespread and ongoing exposure [12]. However in relation to alcohol harm reduction campaigns, with the exception of those specifically focused on drink driving campaigns [13], reviews have concluded that these campaigns are ineffective in changing behaviour [12, 14, 15]. Yet, this conclusion may be premature given there are in fact few high-quality published evaluation studies of such campaigns [16, 17], and there has been little research examining the message characteristics used or whether certain message attributes are more impactful than others [18–20].

Systematic content analysis can provide a useful foundation for understanding mass communication effects and identifying research needs to improve campaign efforts [21]. An early content analysis identified the dominant strategies of drink driving prevention television advertisements (ads), with findings providing a validated typology of drink driving prevention messages that considered dimensions of fear, modelling, positive, informational/testimonial appeal, and empathy strategies [22]. This typology has since been used by other researchers to guide comparative studies of message characteristics in an effort to optimise campaign effectiveness [23, 24]. In other areas of public health, content analyses of tobacco control and obesity prevention ads have defined the scope and range of message attributes commonly used, enabling message-testing studies to examine the effectiveness of key differentiating attributes and providing guidance as to which message attributes have been over- or under-utilised [25–32].

To date, content analyses of alcohol harm reduction ads have been limited to drink driving ads. We anticipate similar themes and message features may have been used in other alcohol harm reduction ads as in drink driving ads, as well as in obesity prevention and tobacco control advertising, since the behaviours of alcohol consumption, unhealthy eating and tobacco use have certain similarities [33–35]. These include an addictive or habitual component to the behaviour, utility in facilitating social interactions and managing negative affect, the presence of strong commercial interests focused on increasing consumption, and the risk of significant long-term health harms.

The overarching aim of this study was to systematically examine the content of English-language alcohol harm reduction ads to identify the message topics, behavioural objectives, emotional tone, and execution elements most commonly used, as well as the audience segments targeted most frequently. By identifying key attributes on which ads vary, this content analysis is intended to guide more rigorous audience-testing research to assess the impact of alternative message characteristics on key audiences and, ultimately, enhance the development of alcohol harm reduction campaigns.

### Message topic

A basic dimension on which alcohol harm reduction ads may vary relates to the key information communicated by the message; that is, the message topic. We aimed to explore the extent to which alcohol harm reduction ads have used five different topics based on the types of messages frequently observed in tobacco control and obesity prevention advertising [26, 30, 31]. Three topics provided reasons *why* to change the behaviour by focusing on (1) the short-term or (2) long-term harms of alcohol consumption, or (3) the facilitators of and negative consequences associated with underage drinking. The fourth topic provided advice on *how* to change the behaviour, and the fifth topic *advocated* for action by increasing public and political support for the implementation of alcohol-control policies.

Based on behaviour change theory, messages that provide reasons *why* the public should change their alcohol-related behaviours are likely to work by influencing viewers’ readiness to act by enhancing their beliefs that the outcome depicted in the message is serious and that they are personally susceptible [36]. Ads may do this by presenting content about the negative health and safety effects of consumption, including both short-term (e.g. injuries, violence) and long-term (e.g. cancer) harms, or alternatively, by focusing on the drivers and negative effects of underage drinking. Messages that aim to increase viewers’ self-efficacy to change their behaviour provide advice on how to change alcohol-related behaviours and present recommended actions (e.g. saying no to the next drink; looking after family and friends while drinking) and/or counter perceived barriers to change [36–39]. By comparison, advocacy messages aim to increase public or political support for alcohol-related policy change either directly or indirectly by prompting policy-related discussions and activities within the community [12, 40, 41]. For example, campaigns could urge viewers to sign a petition to ban alcohol marketing or they could highlight the public’s concern about a particular issue in order to stimulate political attention.

### Behavioural objective

Another way in which alcohol harm reduction ads may vary is in terms of the specific behaviour that is targeted for change or reinforcement [42]; that is, the behavioural objective of the ad. Even though all alcohol harm reduction ads are ultimately designed to contribute to the broad goal of reducing alcohol-related harm, as observed in other areas of public health [13, 43], this broad goal can be achieved through addressing several specific behaviours. For instance, while long-term health harms are primarily influenced by the amount of alcohol consumed by individuals, short-term injuries to the self and others are additionally determined by the way in which individuals behave when drinking. Therefore, while the main behavioural objective of some ads may be to promote reduced personal consumption of alcohol, others may have a major focus on getting drinkers to behave more responsibly or to not be violent when drinking. Furthermore, it is possible for ads with the same topic to be used to achieve different behavioural objectives. For instance, an ad portraying the harms associated with underage drinking may be used to encourage adults to never supply alcohol to teenagers or to inform adults of their influence on underage drinking through role modelling. We therefore aimed to assess the range of behavioural objectives targeted by alcohol harm reduction ads.

### Emotional tone

The elicitation of an affective response is an important pathway through which public health communications can promote positive changes in behaviours [31, 44]. Affect influences human decision making and behaviour in several ways, including by encouraging greater scrutiny of risk information, guiding automatic (largely unconscious) judgements about whether a given behaviour or substance is likely to be healthy or unhealthy, and motivating behavioural action [44, 45]. Emotional responses can be either positively or negatively valenced, and the particular type of emotional response elicited may influence campaign effectiveness. For instance, in obesity prevention, Dixon et al. [27] found ads eliciting strong negative emotional responses performed well on short-term markers of advertising impact. Similarly, within tobacco control, Durkin et al. [46] found that higher levels of exposure to ads eliciting negative emotions were associated with increased quitting behaviours, whereas Richardson et al. [47] found that positive emotion campaigns were most effective at increasing help-seeking through quitline calls, while those with negative emotive content were also found to impact on call rates at higher levels of exposure. Since little is known about the emotional tone employed by alcohol harm reduction ads, we aimed to assess their dominant emotional tone.

### Execution characteristics

The presentation and execution of messages can vary along many dimensions and these can increase the likelihood of acceptance or rejection by the target audience [48–50]. First, we sought to examine the frequency of references to low-risk drinking guidelines within alcohol harm reduction ads. Many governments have developed such guidelines to provide recommendations as to the maximum amount of alcohol that should be consumed to keep risk of harm at a low level [51]. For instance, the Australian Government issued updated national guidelines in 2009 [52] which advised no more than two drinks on any day to stay at a low risk of long-term harm and no more than four drinks on any occasion to minimise the risk of short-term harms; yet, there has been no national effort to promote these guidelines [53, 54]. Upon the recent release of proposed new guidelines for the United Kingdom, the authors recommended mass media campaigns be used to disseminate the guidelines to the public [55]. Since there has been debate over guideline effectiveness [56] and the impact of publicising them has not yet been rigorously assessed [57], we aimed to examine the prevalence of guidelines messages in ads.

Common executional styles used in public health campaigns include personal testimonials, animations, simulations of disease processes or medical procedures, the use of actors to depict a dramatised scenario and the use of experts to convey factual information [26, 28, 31, 58]. Executions that include graphic imagery have been shown to be beneficial in public health campaigns. For example, campaigns depicting visceral images of the physical consequences of smoking have been found to increase quit attempts amongst smokers [59, 60], and a study comparing audience responses to obesity prevention ads found that the top performing advertisement was the one that featured graphic images of fat inside the body [27]. Hence, the present study also aimed to document the dominant executional style and use of graphic representation of negative consequences.

Given that behaviours can be symbolically modelled to audiences via mass media [61], the extent to which health risk behaviours are depicted in public health advertising may also be an important predictor of audience impact. Studies of tobacco control campaigns have produced mixed evidence as to whether depictions of smoking in campaign messages can imply that smoking is a prevalent, normal, and accepted behaviour [28, 62], or stimulate urges to engage in the behaviour [63–65]. Therefore this study aimed to examine the extent to which these ads portrayed alcohol consumption either implicitly (e.g. images of alcohol being purchased but not directly drunk) or explicitly (e.g. images of people drinking alcohol).

Campaigns also vary in terms of whether they are designed for a general audience or a specific target audience based on certain sociodemographic, cultural, or behavioural characteristics [66]. One review of public health mass media campaigns suggested that those focused on whole populations have the greatest impact [12] and provide an economically sustainable approach to generating positive cultural change [67, 68]. However, given age and gender differences in alcohol consumption and experience of harms [1, 52] and gender-specific guidelines for low-risk drinking in some jurisdictions [51], it is reasonable to expect that alcohol harm reduction ads may target particular demographic sub-groups. Campaigns may also target either the individuals whose behaviour they are trying to change, or they may include an empathy appeal [22], whereby the consequences of the behaviour (i.e. drinking) are depicted for people other than the one who enacts the behaviour; a strategy shown to be effective in drink driving campaigns among high-risk populations such as young adults [69]. We therefore sought to assess the extent to which these ads contained content or messages that appeared to target a general or specific audience and either drinkers themselves or the family and friends of drinkers.

Given the levels of alcohol consumption worldwide and the considerable harm attributed to it, public health mass media campaigns offer one way to potentially reduce the burden of alcohol-related harm. In view of the limited knowledge about the advertising characteristics of effective alcohol harm reduction campaigns, the aim of this study was to describe how public education messages on alcohol harm reduction have been presented to date. Identifying the key attributes on which ads vary will guide future rigorous audience-testing research to better inform alcohol harm reduction campaign development.

## Method

### Advertisements

The sample comprised public health ads addressing alcohol harm reduction recently produced in the period 2006 to 2014 inclusive. As television remains the dominant mass medium to reach the whole population (e.g. [70]), we focused on electronic ads that would be suitable for airing on television. Starting in September 2014, English language ads were identified using two main strategies: 1) through exhaustive internet searches of Google and video sharing sites YouTube and Vimeo, using keywords “mass media”, “campaign”, “advertising”, “alcohol harm reduction/prevention”, “anti-drinking/alcohol/binge”, “underage drinking”, and “public education”; 2) specific searches for ads on relevant government and health agency websites and their YouTube channels where available. We excluded ads focused on alcohol in pregnancy due to the limited population such messages are relevant to at any point in time (i.e. approximately only 2% of Australians 15 years and over are pregnant each year [71–73]), and ads focused on drink driving since extensive work has already been done in this area [13]. We also excluded ads funded by an alcohol manufacturer or an alcohol industry Social Aspects/Public Relations Organisation [74] due to research indicating these campaigns are often designed to promote the alcohol industry rather than public health [75–77].

A total of 110 individual ads from 72 different alcohol harm reduction campaigns met the inclusion criteria and were subsequently coded; additional details about the sample of ads are available in Additional file 1: Appendix A. Of the 72 campaigns, 47 were comprised of one ad only, 16 were comprised of two ads (e.g. a male and female version), seven were comprised of three ads, and two campaigns were comprised of four and six ads respectively.

### Coding protocol

The codebook was developed *a priori*, informed by previous work in the drink driving, tobacco, and obesity domains [22, 26, 30, 31, 78], and refined based on pilot-testing with a sub-sample of alcohol harm reduction ads to enhance relevance and specificity. The codebook consisted of the list of variables to be examined and their definitions, along with standardised response options that were to be entered onto the coding form. Each ad was coded against 10 variables: dominant topic, behavioural objective, emotional tone, and seven execution characteristics—presence of guidelines, style, graphic imagery, portrayal of drinking, target audience, gender specific messages, and subject of depicted harms. Table 1 defines each variable and specifies the coding response options; additional details of the codebook are available in Additional file 2: Appendix B.

**Table 1:** Variables used to code alcohol harm reduction advertisements and associated Krippendorff’s alpha coefficients

Variable	Coding response options	Alpha
*Message Topic*		
Dominant topic. What is the key information communicated in the ad?	A. Short-term harms of drinking (depicts short-term harms of drinking e.g. injury, violence, unsafe/unwanted sexual experience, vomiting, social embarrassment etc.) B. Long-term harms of drinking (depicts long-term harms of drinking e.g. cancer, heart disease etc.) C. Underage drinking (depicts dangers of underage drinking or adult role modelling of drinking behaviours for those underage) D. How to change behaviour (depicts how to change alcohol consumption behaviours) E. Advocacy/Policy change (advocates for or aims to influence policy change)	0.96
*Behavioural objective*		
What is the objective of the ad? That is, what is the main behaviour the ad is trying to elicit?	A. Reduce alcohol consumption/limit drinking B. Behave responsibly when drinking alcohol/don’t get drunk when drinking C. Look after friends and family when they are drinking D. Talk to a friend or family member about their drinking E. Limit drinking around minors F. Never supply alcohol to minors G. Parents to talk to their children about alcohol H. Promote policy change	0.91
*Emotional tone*		
Emotional response likely to be elicited among target audience	A. Negative (e.g. fear, anxiety, disgust, worry, shame, guilt, regret, sadness etc.) B. Positive (e.g. hope, inspiration, determination, empowerment etc.) C. Negative and positive D. Neutral (i.e. no emotion)	0.71
*Execution characteristics*		
Presence of drinking guidelines/recommendations to reduce harms	A. Yes B. No	0.96
Style	A. Dramatisation (mini drama scene, with actors depicting a short story) B. Simulation/Animation (involves simulations, animations or models of people, alcohol, or key concepts) C. Factual (presents factual, medical, or statistical information) D. Personal testimonial (depicts a person telling “their story”)	0.96
Graphic imagery	A. Yes (explicit graphic or negative visceral imagery portraying the consequences of drinking alcohol that elicits a visceral “ugh!” response) B. No	0.83
Portrayal of drinking	A. Implicit portrayal only (alcoholic beverages being bought, ordered, held, poured - but not actively being drunk) B. Explicit portrayal (people actively drinking alcoholic beverage/s) C. No portrayal	0.84
Target audience: age or role	A. Children/adolescents (under 18 year olds) B. Specifically young adults (18–30 years) C. Specifically parents D. General adult audience (clearly targeted at adults, but not a specific age/role) E. Government	0.91
Target audience: gender specific message	A. Yes (content/messages directed at females or males) B. No gender specific message/unknown	0.88
Subject of depicted harms/consequences of drinking	A. Personal (focus on consequences of drinking for the target audience of the ad) B. Others (focus on consequences of drinking for people other than the target audience of the ad [e.g. ads that target parents but depict consequences for children]) C. Both personal and others (targets drinkers by showing the consequences of their drinking for themselves and for their friends/family members, or targets friends/family members of drinkers and shows the consequences to both the target audience and drinkers themselves)	0.94

All 110 ads were coded independently by one of the authors (KD) and an experienced researcher not otherwise involved with the project. Initially, coders discussed the codebook and definitions to ensure they had a shared understanding of the operationalisation of each variable and then tested the protocol with four alcohol harm reduction ads that did not qualify for inclusion in the sample (e.g. produced in 2005). To ensure adequate intercoder reliability, coding was first conducted independently in sets of 10 ads (the 110 ads were sorted by country and campaign and every 10^th^ ad was selected for coding, ensuring variation in the ads included in each set of 10). After the first set of 10 ads, intercoder reliability was assessed, differences between the coding outcomes were discussed, and the codebook was refined where necessary. This process was then repeated on a second (every 10^th^ ad starting at the 5^th^ position), third (every 10^th^ ad starting at the 3^rd^ position), and fourth set of 10 ads (every 10^th^ ad starting at the 8^th^ position), at which point Krippendorff’s alpha (for the fourth set of 10 ads) showed an adequate level of agreement and reliability between the two coders for all variables. Finally all 110 ads were independently coded by both coders using the final codebook.

Reliability coefficients for all 10 variables exceeded acceptable levels [79], with values ranging from 0.71 to 0.96 and an average value of 0.89 (Table 1). The variable with the lowest alpha value of 0.71 was retained as it was above Krippendorff’s [79] acceptable level of agreement for exploratory research (0.67) and simple percent agreement between the two coders on this variable was 87%. In cases where the same ad was coded differently by the two coders, they discussed their interpretations after re-watching the ad together until consensus was reached.

### Statistical analysis

Data were analysed using Stata SE 14.2 [80], with individual ads as the primary unit of analysis. We first examined the overall proportion of ads containing each characteristic and then examined whether the proportion containing each characteristic varied across four sub-samples of ads defined by the dominant topic: short-term harms, long-term harms, underage drinking and how-to-change. Too few (*n* = 3) advocacy ads were identified to further examine their content. Fisher’s exact test was used to test for differences in the distribution of each message characteristic variable across the four topics, given chi-square tests were inappropriate as one or more expected cell frequencies was <5 in each test.

## Results

Table 2 shows most ads originated from Australia (40%) or the United Kingdom (26%) and most (89%) were between 30 and 60 s long. The majority (66%) had been aired on free-to-air television, while the remainder had been aired in cinemas or on local cable stations or disseminated through online campaigns.

**Table 2:** Ad sample characteristics

	(*N* = 110) %
*Country of Origin*	
Australia	40
United Kingdom	26
New Zealand	15
United States of America	9
Other^a^	9
*Year* ^*b*^	
2006	7
2007	1
2008	16
2009	9
2010	26
2011	6
2012	7
2013	6
2014	15
*Length of ad*	
< 30 s	5
30 s	39
31–60 s	50
> 60 s	5
*Aired on free-to-air television* ^*c*^	
Yes	66

Due to rounding, percentages may not total to 100

^a^ Other countries: Bermuda (*n* = 1 ad), Canada (*n* = 4 ads), Finland (*n* = 1 ad), Macedonia (*n* = 1 ad), Netherlands (*n* = 1 ad), Singapore (*n* = 2 ads)

^b^ The year of production for six ads (5%) could not be confirmed, although visual and auditory characteristics suggested that they were produced between 2006–2014, and were therefore included

^c^ We identified ads that had been aired on free-to-air television as a proxy measure for general population exposure. Twenty-six of the remaining ads (24%) were disseminated via cinema or online campaigns only, two ads (2%) were shown only on local cable/pay-TV stations, and we were unable to confirm the medium for nine ads (8%)

### Message topic

Just over half (52%) of the ads focused on the short-term harms of drinking and 10% focused on the long-term harms (Table 3). Around one-fifth (18%) focused on the effects of underage drinking and/or on adult role modelling of drinking behaviours for those underage, while 17% of ads included a how-to-change message, and 3% focused on advocacy. Only 10% of ads also included a secondary topic.

**Table 3:** Ad characteristics overall and by dominant topic

	All ads	Ads separated by dominant topic (*n* = 107^a^)				
	(*N* = 110)	Short-term harms (*n* = 57)	Long-term harms (*n* = 11)	Underage drinking (*n* = 20)	How-to-change (*n* = 19)	
	%	%	%	%	%	Fisher’s Exact Test p-value
Behavioural objective						
Reduce consumption	38	33	100	15	47	<0.001
Behave responsibly	33	58	0	5	11	<0.001
Look after others when they are drinking	5	2	0	0	26	0.003
Talk to others about their drinking	3	5	0	0	0	0.686
Limit drinking around minors	2	0	0	10	0	0.073
Never supply alcohol to minors	7	0	0	35	0	<0.001
Parents to talk to their children about drinking	5	0	0	20	11	0.004
Promote policy change	6	2	0	15	5	0.087
Emotional tone						
Negative	74	91	82	75	11	<0.001
Positive	15	0	0	0	89	<0.001
Negative and positive	10	9	18	20	0	0.112
Neutral	1	0	0	5	0	0.467
Execution characteristics						
*Drinking guidelines (yes)*	10	4	82	0	0	<0.001
*Style*						
Dramatisation	87	96	45	90	89	<0.001
Simulation/Animation	8	2	45	10	0	<0.001
Factual	5	2	9	0	11	0.131
Testimonial	0	0	0	0	0	--
*Graphic imagery (yes)*	21	37	0	5	5	0.001
*Portrayal of drinking*						
Implicit portrayal only	28	26	64	30	11	0.022
Explicit portrayal	35	42	36	15	32	0.167
No portrayal	37	32	0	55	58	0.002
*Target audience: age or role*						
Children/adolescents	7	5	0	20	5	0.149
Young adults (~18–30 years)	36	58	0	0	37	<0.001
Parents	18	9	0	60	11	<0.001
General adult audience	35	26	100	20	47	<0.001
Government	3	0	0	0	0	--
*Target audience: gender specific message (yes)*	9	5	45	5	0	0.001
*Subject of depicted harms/consequences of drinking*						
Personal (target audience)	52	49	100	25	63	<0.001
Others	25	7	0	70	37	<0.001
Both personal and others	24	44	0	5	0	<0.001

^a^ *n* = 3 ‘advocacy’ ads excluded, as too few ads were identified to further examine their content

Among ads for which the dominant topic was short-term harm (*n* = 57), depictions of violence, injuries and death were the most common short-term harms depicted (58%), followed by vomiting, urination or passing out in public (28%), social embarrassment or regret (12%), and unsafe or unwanted sexual experiences (11%). Among long-term harm ads (*n* = 11), the most frequently featured harm was cancer (82%), followed by stroke (36%), heart disease (18%), and blood pressure (18%), while 36% included other non-specific ‘serious health problems’. Among underage drinking ads (*n* = 20), 75% depicted the negative effects of alcohol consumption by those underage, 20% included information on the role of adults as role models for drinking behaviour, and 5% included information on both topics.

### Behavioural objective

The objective of most ads was to encourage audiences to reduce their alcohol consumption (38%) or to behave responsibly and/or not get drunk when drinking (33%). Fewer ads focused on behaviours related to others, such as looking after others when they are drinking (5%) or talking to others about their drinking (3%). In addition, a small percentage of ads specifically targeted behaviours aimed at preventing underage drinking, such as limiting the supply of alcohol to minors (7%), encouraging parents to talk to their children about alcohol (5%), or limiting adults’ consumption when in the presence of minors (2%). Only 6% of ads specifically encouraged the audience to act in ways that would promote policy change.

As shown in Table 3, the proportion of ads coded as encouraging particular behaviours varied according to the dominant topic of the ad. All of the long-term harm ads included content encouraging audiences to reduce their alcohol consumption, whereas only a third of short-term harm ads had this objective. Conversely, a higher proportion of short-term harm ads aimed to encourage audiences to behave responsibly when drinking compared with ads with the three other topics. As would be expected, ads addressing underage drinking were more likely to target behaviours regarding the supply of alcohol to minors and parents talking to their children about alcohol compared to the three other topics (Table 3).

### Emotional tone

Across the whole sample, most ads (74%) were coded as attempting to elicit a negative emotional response. However, emotional tone varied according to topic, with short-term and long-term harm ads and underage drinking ads being more likely to have a negative tone than how-to-change ads (Table 3). In contrast, only how-to-change ads featured a positive only emotional tone.

### Execution characteristics

Only 10% of ads included drinking guidelines or recommendations, although these were much more common in long-term harm ads than other ads (Table 3). Overall, the vast majority of ads used dramatisation (87%) rather than simulation/animation (8%) or direct factual information (5%), and no ads used a testimonial execution. There were several significant differences in the use of executional styles across topics. Dramatisations were more prevalent in short-term harm, underage drinking, and how-to-change ads than in long-term harm ads. In contrast, simulation and/or animation of people, alcohol, or key concepts such as chronic diseases was more prevalent among long-term harm ads compared to ads with other topics (Table 3). Around one-fifth (21%) of all ads included graphic imagery, although this was more likely to be used in short-term harm ads than in ads with other topics. Overall, 37% of ads did not portray any alcohol consumption, 28% contained implicit portrayals only (e.g. actor ordering a drink or holding a bottle), and 35% contained explicit portrayals (e.g. actor drinking alcohol). All long-term harm ads portrayed drinking either implicitly or explicitly, and a significantly higher proportion of the long-term harm ads only contained implicit portrayals compared to ads with other topics (Table 3).

Most ads (90%) were coded as containing messages or content targeted at adults; a similar proportion targeted young adults (36%; 18–30 years) and a general adult audience (35%; no specific age target), with fewer ads specifically targeted at parents (18%). Only 7% of ads targeted children and/or adolescents and 3% targeted governments. A higher proportion of short-term harm ads specifically targeted a young adult audience compared to other ads, whereas all long-term harm ads targeted a general adult audience, and underage drinking ads were the most likely to target parents. Most ads (91%) did *not* contain messages or content explicitly targeted towards a specific gender, although long-term harm ads were significantly more likely to contain gender-specific content or messages than were ads with other topics (Table 3).

In the total sample, around half of ads (52%) portrayed personal harms of drinking, 25% showed harms for others (e.g. children in parent-targeted ads), and 24% showed harms for both the self and others. All long-term harm ads depicted the personal harms of drinking, whereas the majority of underage drinking ads depicted the harms for other people. Short-term harm ads were significantly more likely than ads with other topics to show harms for both the self and others (Table 3).

## Discussion

Our comprehensive search for English-language alcohol harm reduction ads suitable for airing on television located 110 such ads from 72 different public health campaigns produced between 2006 and 2014. This indicates that across all English language dominant countries only eight alcohol harm reduction campaigns appeared to have been produced and aired each year on average. One reason for the limited use of alcohol harm reduction campaigns may be the lack of published peer-reviewed research evaluating existing campaigns [16], and among those published evaluations, limited evidence of effectiveness [12, 14, 15]. Encouragingly though, the recent evaluation of an Australian campaign which aimed to increase awareness of the link between alcohol and cancer demonstrated positive impacts of the campaign on drinking attitudes and intentions [81]. In addition, a recent pilot study of young adult drinkers exposed to six alcohol harm reduction ads found subsequent reduced urge to drink compared to young drinkers who had been exposed to six alcohol promoting or non-alcohol product ads [82]. Further peer-reviewed publications of campaign evaluations and/or experimental studies are required to provide more rigorous evidence about campaign effectiveness; such literature will be critical for encouraging greater investment in alcohol harm reduction campaigns. In addition, it is probable that the relatively small number of public health campaigns could have made only a very small contribution to redressing the information asymmetry in alcohol-related media content when considering the vast amount of pro-alcohol content disseminated by the alcohol industry [6, 8]. Restrictions on alcohol marketing have been recommended by public health agencies, researchers, and advocates, including the World Health Organization [83], as a cost-effective intervention to reduce harms caused by alcohol and to help redress the current imbalance in alcohol-related content in the public communication environment [57, 84–86].

The majority of alcohol harm reduction ads identified focused on the short-term harms of alcohol use. Fewer ads focused on the long-term harms, underage drinking, giving advice as to how to change drinking behaviours, or on advocating for policy change. Given that about 70% of all alcohol-attributable disability-adjusted life years globally are due to long-term harms, including cancers, cardiovascular diseases, neuropsychiatric disorders, and infectious diseases [1], the emphasis within campaigns on the short-term harms of drinking is disproportionate to disease burden, but rather may reflect social and political concern around these highly visible and newsworthy alcohol use consequences that are experienced disproportionately by younger people [52]. At the same time, the smaller number of ads addressing long-term harms may reflect the fact that the epidemiological evidence linking alcohol to these conditions is still emerging [87], that it is harder to attribute these diseases and conditions to alcohol alone, and that these harms are less conspicuous and harder to depict visually than many of the short-term harms. Future alcohol harm reduction campaigns may benefit from a greater focus on messages communicating these long-term harms. These messages could improve public understanding of the long-terms harms of alcohol use, which is currently low [88], enabling drinkers to make more informed decisions about their alcohol consumption. Furthermore, given that a large proportion of the drinking population consumes alcohol in a manner more likely to cause long- rather than short-term harm [1], such messages might be more broadly relevant or motivating. While some research suggests that audiences may respond more strongly to messages regarding short-term consequences as the risk feels ‘close’[89] and it is easier to discount messages about effects not likely to be felt in the near future [90], other research has demonstrated messages focused on serious long-term harm consequences are effective in changing smoking-related attitudes, intentions, and behaviours [30, 31], including among young people [91, 92]. Future research should aim to examine the impact of long-term harm alcohol harm reduction ads and investigate their effectiveness across various segments of the population.

One-third of all ads encouraged audiences to behave responsibly when drinking and/or to avoid getting drunk. This focus on responsible behaviour when drinking is important to the extent that many of the short-term harms, and much of the alcohol-attributable harm to non-drinkers, arises from the ways in which drinkers behave when they become intoxicated. However, one concern with such messages is that definitions of “responsible drinking” or “responsible behaviour” often lack clarity and specificity, and so are open to individual interpretations [93]. Just over one-third of ads specifically aimed to encourage reduced alcohol consumption (including 100% of ads focused on long-term harms). In addition, despite the development of drinking guidelines by many governments [51], such guidelines were rarely mentioned. Taken together, these findings highlight an opportunity for more alcohol harm reduction campaigns to focus specifically on encouraging reduced consumption, in part through the communication of low-risk drinking guidelines and defining “responsible drinking”. Given the latest epidemiological evidence suggests that *any* alcohol consumption increases the risk of harm in both the short- and long-term [52, 94], and there is reason to expect that drinking guidelines have the potential to shift social norms about acceptable levels of drinking by providing a low-risk ‘anchor’ for drinkers [95], an increased use of such messages may be an important step towards reducing harms caused by alcohol. In addition, incorporating guideline messages into future alcohol harm reduction campaigns may provide an avenue through which campaigns can indirectly increase public and political support for complementary policies that aim to reduce alcohol consumption [96].

Most alcohol harm reduction ads were coded as aiming to elicit negative emotional reactions. The how-to-change ads were the only ones to employ a positive only emotional tone. Future research could compare the impact of negatively and positively valenced alcohol harm reduction campaigns to examine whether the greater persuasive impact of negative emotion ads observed in other domains [27, 30, 97, 98] also applies to alcohol harm reduction. In this regard, Stautz and colleagues’ recently published pilot study suggested that the impact of exposure to alcohol harm reduction advertising on reduced urge to drink among young adults was fully mediated by the elicitation of negative affect, although behaviour change was not studied as an outcome [82]. Larger scale studies with younger and older drinkers are needed, with follow-up behavioural outcomes assessed. There is scope for rigorous experimental tests of negative and positive messages alone versus in combination, and for the general population versus high-risk drinkers, which could provide valuable insights for campaign planning.

In general, the majority of ads employed dramatisation, except among ads depicting the long-term harms where an equal number of ads used dramatisation and simulated imagery of the internal harms caused by alcohol. Some indicative tobacco control research has suggested that graphic dramatisation of health harms may be more effective than simulations or animations, such that future long-term harm campaigns may benefit from an increased use of dramatisations [99]. Our study found no ads employing a testimonial execution style, which has been used effectively in road safety [22, 100] and tobacco control campaigns [30, 46]. Testimonial messages can increase feelings of personal vulnerability and urgency for taking action, and reduce counter-arguing [101], and therefore represent another potential opportunity for enhancing the effectiveness of future alcohol harm reduction campaigns.

It is notable that 35% of alcohol harm reduction ads were designed to appeal to a general whole-of-population audience (including 100% of ads with a focus on long-term harms), while 36% targeted young adults specifically. Future research should investigate the effects of general versus youth-targeted ads on youth and younger adults, given that evidence from tobacco control suggests that adult-targeted campaigns can also exert positive effects on younger audiences [102, 103], and as such may represent a more cost-effective approach for reaching large segments of the population with a more unified message [68].

There was a notable lack of alcohol harm reduction ads produced in the United States (US) relative to Australia and the United Kingdom, especially considering that the scale of alcohol-related harm is similar in the US to those countries where most ads were produced [1]. Even in the absence of national campaigns in the US, it is surprising that there has also been such little activity at the state and community levels, given a recent study found that is where many obesity prevention campaigns originated [104]. Furthermore, six of the ten ads from the US identified in our study focused on underage drinking—either by demonstrating the harmful effects on youth or encouraging parents to talk to their children about drinking—indicating a need for additional campaign efforts to inform the broader American public of the short- and long-term harms associated with alcohol consumption.

### Limitations and strengths

Our sample was limited to publicly available English-language ads suitable for television, and so may not reflect the characteristics of ads produced in other languages or educational messages produced for other advertising media, such as radio, poster and point-of-sale campaigns. Although a thorough search was conducted, it is possible that other ads exist. It should also be noted that this study focused on documenting aspects of message content that previous research has identified as important to the potential persuasiveness of public health advertising, rather than assessing public reactions to such advertising content. We cannot be certain that the coders’ appraisals of the emotional tone of the ads reflect the actual emotional responses elicited in the audience. Furthermore, our assessment of each ad’s behavioural objective and target audience was inferred based on the language and imagery used in the ads; we could not access information on the behaviour that was intended to be elicited by the producers of each ad, the audiences they intended to reach, or for whom the media buys for these ads were targeted.

It is important to note that only two-thirds of the ads included in this sample had definitely been aired on free-to-air television, while other ads had been aired in cinemas or on local cable stations or disseminated through online campaigns. The number of ads produced (and identified in our search) is indicative of the level of investment in producing alcohol harm reduction public health advertising, but may not reflect population exposure to this advertising (e.g. substantial exposure could be achieved through a small number of ads aired frequently). However, it was beyond the scope of our content analysis to collect objective measures of advertising exposure through target audience rating points or data on advertising expenditure, so we are unable to quantify the extent of investment in and public exposure to alcohol harm reduction campaigns that occurred over this 9 years period. While the results of this content analysis are descriptive, they provide an essential foundation for theoretical development and hypothesis-testing experimental research to explore the effectiveness of different message characteristics for different segments of the population.

## Conclusions

This study appears to provide the first detailed examination of the content of alcohol harm reduction ads internationally. We applied a rigorous coding procedure and achieved high inter-rater reliability to generate a robust characterisation of ads. We conclude that alcohol harm reduction ads produced between 2006 and 2014 have employed some message attributes much more frequently than others, including a focus on short-term harms, an emphasis on encouraging reduced consumption or responsible behaviour when drinking, an emotional tone that is predominantly negative, and dramatisations rather than other executional styles. The findings from this study should inform subsequent research aimed at identifying which of these message characteristics are most effective at reducing harmful drinking behaviours. For example, the message characteristics data could be used to better understand the patterns of responses observed in message rating or experimental studies of the immediate impact of alcohol harm reduction advertisements [82]. Future research could also use the information generated from this content analysis in conjunction with advertising exposure data (e.g. targeted audience rating points) to provide estimates of the frequency of population exposure to certain campaign characteristics (e.g. [58, 104]). To examine associations with campaign outcomes, the message characteristics identified in this content analysis could also be used to assist interpretation of the results of population survey or cohort studies of changes in drinking behaviours following exposure to campaign messages with different characteristics [21]. Ultimately, we hope such research will lead to the development of more successful alcohol harm reduction campaigns internationally.

## Additional file

Additional file 1: 
**Appendix A.** Details and synopses of 110 alcohol harm reduction advertisements. This file contains a detailed list of the 110 alcohol harm reduction ads used in this content analysis. For each advertisement the following details are included: ad name, campaign name, country of origin, sponsor, ad length and a brief synopsis. (PDF 56 kb)
Additional file 2:
**Appendix B.** Alcohol harm reduction advertising classification codebook. This file contains the detailed codebook used to classify alcohol harm reduction ads. The codebook consists of the list of variables examined and their definitions, along with the standardised response options. (PDF 65 kb)

## Abbreviation

ad: Advertisement
