# Supplementary material for: Alcohol harm reduction advertisements: a content analysis of topic, objective, emotional tone, execution and target audience
Source: BMC Public Health. 2017 Apr 11;17:312. doi: 10.1186/s12889-017-4218-7 (PMC5387386; doi:10.1186/s12889-017-4218-7)
Supplement: Additional file 1: — Appendix A. Details and synopses of 110 alcohol harm reduction advertisements. This file contains a detailed list of the 110 alcohol harm reduction ads used in this content analysis. For each advertisement the following details are included: ad name, campaign name, country of origin, sponsor, ad length and a brief synopsis. (PDF 56 kb) [file 12889_2017_4218_MOESM1_ESM.pdf]

APPENDIX A: Details and synopses of 110 alcohol harm reduction advertisements

Ad name	Campaign name	Country	Sponsor	Length (s)	Synopsis
Magic Pancakes		Australia	adShame	60	This ad features animated scenes from a children's program of a wizard and a unicorn who are excited to make magic pancakes, but are interrupted by footage from alcohol promoting ads. If alcohol advertising doesn't belong during cartoons, why is it okay during live sport before 8.30pm when thousands of young children watch. No booze ads before 8.30pm. No exceptions.
Is your child's club a Good Sports club?		Australia	Australian Drug Foundation	70	Playing a game of football in the backyard, two young boys celebrate by chanting "let's get wasted" and "cheers" before they skol cups of water. They pretend to urinate on the fence using water bottles, abuse each other and fight, vomit, sing beer songs and pass out on the grass. The mother walks outside looking concerned, "Is your child's club a good sports club?".
Be the influence - tackling binge drinking		Australia	Australian National Preventive Health Agency, Australian Government	30	Australia's leading sports organisations have made a pledge to tackle youth binge drinking. The ad features Australian sports stars from netball, football, swimming, basketball, baseball, soccer and surfing. "It's all about coming together to have a positive influence on our drinking culture. We are strong enough to say enough is enough, are you? Be the influence."
Boy	Can't rewind your actions	Australia	Central Institute of Technology and City of Perth	50	Three young males are drinking at home, and then go to a pub where they are refused entry. They continue to loiter on the street and receive a written warning by police to move on. One youth punches a policeman and is handcuffed. The ad ends with the tagline "you can't rewind your actions".
Girl	Can't rewind your actions	Australia	Central Institute of Technology and City of Perth	45	Three young women are drinking and getting ready for a night out. At the nightclub on a crowded dancefloor one woman keeps bumping into another group, who eventually yell at her to dance somewhere else and then smash a bottle over her head. We then see the girl's evening rewinding back to the beginning of the night, with the tagline "you can't rewind your actions".
Boy	Don't turn up drunk	Australia	Central Institute of Technology and City of Perth	45	Young adults are drinking and having a good time, and go to a nightclub where one male is refused entry. The police arrive as he is trying to push his way in. The cost of the night is calculated during the ad (beer, taxi, criminal record for obstructing police). The ad ends with the male in jail and the tagline "you decide the cost".
Girl	Don't turn up drunk	Australia	Central Institute of Technology and City of Perth	40	A group of young adults are drinking and then catch a taxi to a nightclub. The young women jump the queue to enter the nightclub. When challenged they become aggressive, and one of the women punches another. The cost of the night is calculated during the ad (beer, taxi, criminal record for assault, ambulance service). The ad ends with the tagline "you decide the cost".
Coffee table / Pub fight	Don't turn a night out into a nightmare	Australia	Commonwealth Government	60	Young people are drinking and celebrating at a house party when a female falls onto a glass coffee table and cuts her hands. In the second scene, a group of friends at a pub order rounds of drinks and a fight breaks out as one of them is bumped by a man walking past. The ad ends with the tagline "don't turn a night out into a nightmare".
Party bushes / Pedestrian accident	Don't turn a night out into a nightmare	Australia	Commonwealth Government	60	Young people are drinking at a house party, a couple become intimate in the backyard and others take photographs and laugh. In the second scene, a young male takes beers from home and drinks them with friends at the park, walking along a road he stumbles and is hit by a car. The ad ends with the tagline "don't turn a night out into a nightmare".
Summer Harms		Australia	Foundation for Alcohol Research & Education / NSW and ACT Alcohol Policy Alliance	80	In this ad text is displayed over background photos. This summer we'll enjoy long hot days, celebrations, barbies with friends. We'll visit 15,100 liquor outlets and consume 14M litres of alcohol. The result… 7,800 assaults, 3,000 domestic violence incidents, 12,300 hospitalisations, and 300 deaths. A heavy summer alcohol toll. Premier O'Farrell, there is a solution. www.naaapa.org.au
A coward's punch can kill		Australia	Individual (Danny Green)	30	A young man spills his beer in a pub as another man pushes past and bumps into him. He gets up to throw a punch but is interrupted by boxer, Danny Green, who says "one punch thrown in anger, without warning, without gloves, can be deadly. It can end his life and ruin yours". The ad ends with the tagline "a coward's punch can kill".
Too much booze, we all lose		Australia	National Drug Research Institute and Kalgoorlie Alcohol Action Project	45	Against a soundtrack of Christmas carols, a group of young adults are drinking alcohol at home before going to the pub. While some drink water, the main character refuses when offered. He harasses a woman at the pub. As he is thrown out by the bouncer he knocks over another woman who requires medical attention, an he ends up being arrested.
Aggressor	Stop before it gets ugly	Australia	New South Wales Government	30	A young man ordering drinks at the bar is approached by another man with a bloodied face who says "later on you'll take something I say the wrong way and hit me causing a brain haemorrhage. You'll get put away for years, you'll lose your job, your girlfriend, your mates." The ad ends with the tagline "stop before it gets ugly".
Influencer	Stop before it gets ugly	Australia	New South Wales Government	30	When a young man buys another round of drinks for his friends, another man sits down in his seat. He is injured and bleeding and explains to the friends that he is the guy their mate is going to punch later. He says they can stop it happening by helping the friend to pace himself or taking him home early. The man returns with the drinks to find his friends looking uncomfortable. The ad ends with "stop before it gets ugly".
Know when to say when		Australia	New South Wales Government	30	A series of scenarios are used to ask the question 'when have you had enough?', by depicting adult drinkers knocking over a glass, messing up a cab, starting a fight with friends, waking the neighbours, entering a booze bus etc. The ad finishes with the tagline "know when to say when".
Lasso	Champion moves	Australia	Northern Territory Government	30	A group of young men realise they are missing one of their friends. Eventually they find him stumbling around a bus shelter. They grab him in a "lasso" move and all head home together. The voiceover ends with "it takes a real champion to keep their mates out of trouble, have you got the moves?".
Muzzle	Champion moves	Australia	Northern Territory Government	30	Standing at the bar in a pub, one man is verbally harassing another man. As the situation escalates, the man's friend steps in to diffuse the situation by putting his hand over his friend's mouth like a muzzle and saying that "he just doesn't know when to shut up". The voiceover states "it takes a real mate to keep their friends out of trouble".
Receptionist	Champion moves	Australia	Northern Territory Government	30	A young man is giving unwanted attention to a woman at a nightclub. As her boyfriend arrives looking angry, the man's friend intervenes and pretends he has a phone call for the man, in order to diffuse the situation and get his friend to leave. The voiceover states "it takes a real mate to keep their friends out of trouble".
Not one more		Australia	NSW and ACT Alcohol Policy Alliance	45	The ad features interviews with prominent experts. Each starts with the line "Not one more…" "kid's life derailed" "alcohol related hospital admission" "parent told their child is never coming home" "alcohol related death" "community without a voice" "family living in fear" "child with a Fetal Alcohol Spectrum Disorder". The ad ends with "one more injury or death in NSW from alcohol is one too many".
Alley	Every drink counts	Australia	Queensland Government	45	At a nightclub where young people are drinking, we see a young woman become intoxicated as she consumes more and more alcohol. As the night goes on, the image becomes blurry and then we see the young woman leave with a group of men. In the next scene, she is lying on the footpath with the men standing over her, who then run away. The ad ends with "every drink counts".
Crash	Every drink counts	Australia	Queensland Government	45	Drinking, dancing and playing pool with friends, a count on a young man's t-shirt shows the number of drinks he's had. He is eventually escorted out of the bar by a bouncer, and the count is showing 15. Waiting in a taxi line, his friends find him and grab him, but he falls onto the road in front of a passing car and is run over. The tagline is "every drink counts".
Angie		Australia	Queensland Government	30	When offered another drink, Angie says no. She then visualises all the things she could do such as meditating, hosting a TV show, saving a child from in front of a bus, swimming in a race for Australia and being a kung-fu master. The ad ends with the tagline "it feels good to say no when you want to say no".
Don't kid yourself		Australia	Queensland Government	30	A young girl is being assaulted in an alleyway. Going backwards in time, we see her leaving a party with two young boys after enjoying herself and dancing. Rewinding even further we see her preparing for the party with friends, and before that we see her leaving her house where her father has provided her with a slab of alcoholic drinks.
Daughter	Drink too much, it gets ugly	Australia	South Australia Health	15	A man vomits into a toilet bowl. His young daughter walks in and asks if he is okay. He replies "Daddy isn't feeling too well this morning" and shoos her out of the bathroom as he vomits again. The ad ends with the tagline "drink too much, it gets ugly".
Nightclub	Drink too much, it gets ugly	Australia	South Australia Health	30	Waiting in a line outside a nightclub, the main character looks some women up and down who call him a pig and disgusting. As he gets to the front of the queue the bouncer refuses him entry. He argues back but then trips over the barrier rope. The ad ends with the tagline "drink too much, it gets ugly".
Ambulance	Drink too much, you're asking for trouble	Australia	South Australia Health	17	In the back of an ambulance a young girl is lying on the stretcher. Using a suction hose, the paramedic sucks some of the vomit from her mouth so it doesn't go into her lungs and suffocate her, and helps her lean to the side to vomit into a bag. The ad ends with the tagline "drink too much, you're asking for trouble".
Daughter	Drink too much, you're asking for trouble	Australia	South Australia Health	17	A man vomits into a toilet bowl. His young daughter walks into the bathroom and asks if he is okay. The voiceover states "to seek help for you and your family, go to alcohol.sa.gov.au or call 1300 13 1340". He shoos her out of the bathroom as he vomits again. The ad ends with the tagline "drink too much, you're asking for trouble".
Friends	Drink too much, you're asking for trouble	Australia	South Australia Health	17	Two girls are in the bathroom of a nightclub when they notice a girl on the floor in one of the toilet cubicles with her head resting on the toilet seat. Giggling, one recognises the drunk girl and says she works in her office, and that she can't wait to tell everyone this story on Monday. The ad ends with the tagline "drink too much, you're asking for trouble".
Paddy Wagon	Drink too much, you're asking for trouble	Australia	South Australia Health	17	Two men, one with blood on his shirt, are in the back of a Police van. The following conversation takes place between the two: "Did you need to smash the guy? He was only being a tool" "I was drunk alright" "We're going down for this Richo". The ad ends with the tagline "drink too much, you're asking for trouble".
Step Back Think		Australia	Step Back Think	80	Three young men come out of the pub to hail a taxi and notice a drunk guy staggering around trying to use his phone. One asks the drunk man "one too many or your first?" and he tells them to "piss off" and the young man becomes angry and punches the drunk guy, knocking him to the ground. When he doesn't move they run off. We see the young man looking scared with the text '8 years in prison', while a friend tries to revive the drunk guy '8 hours to live'.
Think before you drink		Australia	Tasmanian Drug Education Network	30	Children act out a scene, voiced by adults, where they are having a few drinks and talking about being worried about their kid's drinking. As the boy goes to leave on his scooter, the girl asks if he is alright to drive, he replies "yeah, I can hold my drink". The ad ends with "what are you teaching your children?".
Marathon Millie	No excuses	Australia	VicHealth and Victorian Government	30	Millie refuses a tequila shot her friend gives her saying she is done for the night. When her friends questions her, Millie makes up an elaborate excuse that she has an early morning tomorrow and appears in running gear saying her race begins at 7am if her friends wants to watch. The ads ends with tagline "you don't need an excuse", promoting the idea that it's ok to drink without getting drunk.
Snake-eye Stevie	No excuses	Australia	VicHealth and Victorian Government	30	Two men are at a pub. When one of the men is offered another beer by his friend he replies "no, I can't. I'm on antibiotics". The friend questions him and so the man makes up an elaborate excuse of having 'snake eye'; as he tells this lie, we see a snake wrap itself over his shoulder. The ads ends with tagline "you don't need an excuse", promoting the idea that it's ok to drink without getting drunk.
Wingman		Australia	Victorian Government	24	This ad features AFL coaches asking the question "we have wingmen on the field on game day, who is your wingman off the field?". "Being a wingman means keeping your friends safe when out having a good time". Like our Facebook page and take the pledge to be a wingman.
Could happen to you	Alcohol and cancer	Australia	Western Australian Government Drug and Alcohol Office	30	A factual interview with Professor Ian Olver is alternated with simulated imagery from the Australian 'Spread' ad of alcohol causing damage in the body. The ad ends with Professor Olver stating "no matter who you are or what you drink, your risk of cancer increases the more you consume".
Spread	Alcohol and cancer	Australia	Western Australian Government Drug and Alcohol Office	30	A glass of red wine is knocked over and the spilled wine spreads throughout a simulated image of a female body. The voiceover states "with every drink the risk of cell mutations in the breast, bowel, liver and throat increases; these cell mutations are also known as cancer".
Stain	Alcohol and cancer	Australia	Western Australian Government Drug and Alcohol Office	30	The ad shows an empty wine bottle and we see red wine stains on the table. As the camera pulls back we see that the stains spell the word "cancer". The voiceover explains that alcohol can cause cancer which "can develop in the breast, liver, bowel, mouth or throat".
Cogs		Australia	Western Australian Government Drug and Alcohol Office	30	A simulation of moving cogs in an adolescent brain is shown as the voiceover tells us "as a parent you should know that alcohol can affect your child's developing brain, not only their problem solving skills and school performance but also their body, mood, and mental health. Under 18, no alcohol, the safest choice".
Do something about drunkenness		Australia	Western Australian Government Drug and Alcohol Office	30	At a backyard barbeque party we see a man consuming beer. Later on he is dancing inside the house but trips on the rug and loses balance, crashing into a pregnant woman who is knocked against the kitchen bench. In the next scene, a doctor tells the woman that she has lost the baby. The ad ends with the tagline "we can all do something about drunkenness".
Here's to		Australia	Western Australian Government Drug and Alcohol Office	45	Different scenarios depict the diverse ways in which drunkenness affects the whole community, such as bartenders being abused, people living in fear of assault, community members cleaning up rubbish and vandalised property, police bearing bad news, parents worrying about their children. The ad ends with the voiceover "let's keep alcohol under control".

I see		Australia	Western Australian Government Drug and Alcohol Office	45	Watching TV on the couch, an adolescent boy asks his father if he and his friends could "grab a few beers". The father replies that "yeah, can't see the harm". The following scenes depict the harms of underage drinking as witnessed by a taxi driver, school psychologist, paramedic and surgeon. The ad ends with "no one should supply alcohol to under 18s".
Tolerance of drunken behaviour		Australia	Western Australian Government Drug and Alcohol Office	30	A young male is feeling guilty about a friend undergoing physical therapy to walk again after a night at the pub (cause of injury is unclear). The injured man tells his friend "you did nothing", implying he wasn't the cause of the injury, but the friend seems to think he should have intervened to stop the drunkenness. The ad ends with the tagline "putting up with drunkenness can have serious consequences".
What you can't see		Australia	Western Australian Government Drug and Alcohol Office	30	As a man opens a beer at home we see the 'toxic effects' alcohol can have on the heart, liver, bowel and brain through a series of animated anatomical diagrams. "It's what you can't see that can cause the most damage".
Don't drink and drown		Australia	Western Australian Royal Life Saving	30	The ad alternates between showing a young man dancing at a party and him making similar movements under water. The voiceover says "alcohol gives you confidence and makes you more uncoordinated, which you can deal with... unless you are in the water. Don't drink and drown". The ad ends with the man in the water not moving.
Youth and alcohol don't mix		Bermuda	Department for National Drug Control	60	A colourful animated ad that informs parents why children should wait until 18 years of age before drinking (alcohol increases the risk of poor grades, depression, violence and sexual activity), how the parents' drinking can influence children, and that adults should never provide alcohol to a minor.
Hannah	What else got wasted	Canada	Government of Saskatchewan	33	Teenagers around a bonfire are drinking alcohol, dancing and laughing. A couple of girls try to encourage Hannah to join back in with the group but she says no. Hannah then appears to be sick and heads into the surrounding bush. "Hannah passed out from alcohol poisoning. Her friends couldn't find her." The ad ends with the tagline "what else got wasted?".
Justin	What else got wasted	Canada	Government of Saskatchewan	33	Teenagers around a bonfire are drinking from beer kegs, dancing and laughing. Two boys are side by side, arms around each others' shoulders, talking and laughing, when one falls backwards into the fire. "Justin got severely burned. He was only fooling around." The ad ends with the tagline "what else got wasted?".
Female	You always have a choice	Canada	Government of Saskatchewan	30	At a party at the end of the evening a young female has to choose between a cup of coffee or a shot of tequila. The voiceover states "you can stop or go on drinking, remember you always have a choice". The next scene shows the female sleeping on a couch and we hear a telephone message from her boss telling her she is fired for being late again.
Male	You always have a choice	Canada	Government of Saskatchewan	30	A designated driver asks his friends if they are ready to leave, however a young woman interrupts asking who will buy her a drink. The young men look at each other and the voiceover states "go home or keep drinking, remember you always have a choice". In the next scene, a doctor asks the young man for a list of all his recent sexual partners.
Monsters		Finland	Fragile Childhood and A-Clinic Foundation	50	A series of scenarios portray children looking confused and scared. As the camera pans out we see a grim reaper watching a boy in a playground, a girl holding the hand of a zombie, an evil clown watching a boy play soccer and a burglar putting a seatbelt on a boy. The ad ends with tagline "how do our children see us when we've been drinking?".
Don't let alcohol abuse you		Ireland	Ireland Health Promotion Unit	30	A young woman is woken up by a voice coming from an empty wine bottle on the bedside table. The bottle says "where is lover boy now? Forgotten what happened? He hasn't and he is bragging right now". As the girl gets upset and tries to get dressed the bottle mockingly laughs at her. The ad ends with the tagline "don't let alcohol abuse you".
Underage drinking		Ireland	Ireland Health Service Executive	40	As a father deposits alcohol bottles for recycling, his son says "I see his drinking but he hasn't a clue about mine". "You can always find someone to buy it" says a boy outside a bottle shop, and a girl says "if I talk to my parents about drinking they'll just get suspicious". The ad ends with "the time to do something about underage drinking is before it's a problem".
Alcohol destroys		Macedonia	Government of the Republic of Macedonia	30	As a young couple leave a club smiling and laughing and continue down they street, the man runs into an invisible object. As the camera pans out we see he is trapped inside a large wine bottle. The ad ends with the tagline "do not lock your soul in a bottle, alcohol destroys".
Protect your growing children		Netherlands	Trimbos Instituut	30	Young children are trapped inside empty alcohol bottles and glasses in different scenarios including at school, on the soccer pitch and in a living room. The voiceover states "a child only grows up once, alcohol consumption inhibits that growth. It slows physical, mental and brain development. Protect your growing child from alcohol damage".
Kat	Ease up	New Zealand	Government Health Promotion Agency	60	A friend talks to her colleague, Kat, in the tea room at work about how she is worried about Kat's drinking. We see scenes of Kat's alter egos when she is drinking: backstab-Kat, you're-boring-Kat, and fall-down-Kat. The ad finishes with the voiceover 'however you do it, tell them to ease up on the drink".
Matt	Ease up	New Zealand	Government Health Promotion Agency	60	Matt is woken up by his wife, who says "I'm not here to tell you to stop having a great time, but I don't want you to hang out with those mates of yours" and we see scenes of intoxicated Matt's 'mates/alter egos': abuse-your-friends-Matt, money-wasting-Matt. The ad ends with the voiceover "however you do it, tell them to ease up on the drink".
Sam	Ease up	New Zealand	Government Health Promotion Agency	60	Before a ruby match a friend asks Sam if he is coming on the weekend and to bring some meat for the barbeque, a few brews, but not his mates. We see Sam's intoxicated 'mates/alter egos': shouty-Sam, punchy-Sam, hit-on-everyone's-wife-Sam, almost-got-arrested-Sam. The ad ends with the voiceover "however you do it, tell them to ease up on the drink".
Gary	It's not the drinking - It's how we're drinking	New Zealand	Government Health Promotion Agency	45	At a wedding, Gary watches as his alternate self grabs another beer and embarrasses his daughter by insisting that she dance with him. As the daughter walks away embarrassed, alternate Gary tries to breakdance and hurts his back. Gary just shakes his head. The ad ends with the tagline "it's not the drinking, it's how were drinking".
Munro	It's not the drinking - It's how we're drinking	New Zealand	Government Health Promotion Agency	45	Munro sees his alternate self order another beer and proceed to spill it on a woman, interrupt a friend playing pool and ask his boss why he didn't get a pay rise. After arriving home with his girlfriend, she abruptly leaves as he had fallen asleep on the bed. The ad ends with the tagline "it's not the drinking, it's how were drinking".
Rosie	It's not the drinking - It's how we're drinking	New Zealand	Government Health Promotion Agency	45	Rosie sees herself outside the restaurant she is at. She follows her alternate self to a nightclub and watches with embarrassment as she has drinks shots, dances, kisses a work colleague and leaves the nightclub with him. As the taxi drives off Rosie yells "what are you doing?". The taxi pulls over, and the alternate Rosie leans out to vomit.
Danny	It's not the drinking - It's how we're drinking	New Zealand	Government Health Promotion Agency	45	A man drinking with friends at the pub becomes progressively drunk and turns violent toward bar staff. He wakes up in a bloody mess on the bathroom floor and sees his young daughter's horrified face. The ad ends with the tagline "it's not the drinking, it's how we're drinking".
Lisa	It's not the drinking - It's how we're drinking	New Zealand	Government Health Promotion Agency	45	A women is enjoying after work drinks at a pub with colleagues. As the night progresses we see her become more intoxicated. As she leaves the pub a man aggressively takes hold of her dragging her down an alleyway. The ad ends with the tagline "it's not the drinking, it's how were drinking".
Uncle	It's not the drinking - It's how we're drinking	New Zealand	Government Health Promotion Agency	45	A man at a barbeque is playing with children by spinning them through the air. We see him consume numerous drinks through the afternoon and evening. Later on, when a child asks to be spun around again, he obliges in the living room but loses control and throws the child into a cabinet. The man is told by his family to get out, and he leaves appearing remorseful and very intoxicated.
Add nothing	Not beersies	New Zealand	Government Health Promotion Agency	30	Classical music is playing. Water is poured from a beer tap into a beer glass labelled 'not beersies'. "How do you brew a beer as pure as this? We only use the freshest water from super-clean waterfalls, rainclouds, icebergs and the finest kitchen taps and don't add yeast, malt or hops. Not beersies, zero carbs, zero craft, zero beer."
Feel the freshness	Not beersies	New Zealand	Government Health Promotion Agency	30	Classical music is playing. Water is poured from a beer tap into a glass labelled 'not beersies'. "See how it pours, see how it swirls, you can almost feel the freshness, almost taste it, which is strange because water doesn't have a taste, not like orange juice or soup. Not beersies, people drink it with beersies...and sports like karate."
No rules	Not beersies	New Zealand	Government Health Promotion Agency	30	Rock music is playing. Water is poured from a beer tap into a beer glass labelled 'not beersies'. "You don't follow the rules, so why should your beer have to? You don't think inside the box, so why should your beer have to? You don't look or taste like beer, so why should your beer have to? Not beersies, the beersie that isn't a beersie".
Not beer	Not beersies	New Zealand	Government Health Promotion Agency	30	Classical music is playing. Water is poured from a beer tap into a beer glass labelled 'not beersies'. "What is it that sets this beer apart from all others? Maybe it's the crisp refreshing taste, maybe it's the purity, or maybe it's because it's not even actually beer but is water. That's it, that's what it is. Not beersies, the beersie that's water".
Since when	Not beersies	New Zealand	Government Health Promotion Agency	30	Classical music is playing. Water is poured from a beer tap into a beer glass labelled 'not beersies'. "Since when did beer have to toe the line? Play the game? Work for the man? Since when did beer have to taste like beer, or for that matter have beer in it? Since 1990-never. That's when. Not beersies, the beer that doesn't have beer in it".
The secret	Not beersies	New Zealand	Government Health Promotion Agency	30	Classical music is playing. Water is poured from a beer tap into a beer glass labelled 'not beersies'. "What's the secret to great beer? We use the freshest purest water, a perfect blend of oxygen and hydrogen atoms and mix it with nothing, nothing but love. Maybe take a not beersie break or don't. Up to you. It's cool either way."
#Cooldad		New Zealand	Government Health Promotion Agency	45	A dad arrives home to find teenagers partying at his house, he acts 'cool' by using slang language, dancing, and handing out beers, while the teenagers ignore him or are embarrassed. The voiceover states "it is now illegal to give under 18s alcohol without parental consent. Don't be a 'cool' dad, see alcohol.org.nz for how the new law impacts you."
Say yeah, nah		New Zealand	Government Health Promotion Agency	60	A man says "yeah, nah" when offered another beer. When his friend questions him he says "yeah I'm still going to have a good night but nah I don't want another beer" and we see through the friend's eyes his initial disappointment and then the fun they continue to have without drinking any more.
Nails	When to stop	Singapore	Government Health Promotion Board	60	An older woman is being interviewed about her extremely long fingernails: "long nails are beautiful, I love them so much, it becomes part of my life, they share my happiness and my sorrows, you see I'm so proud of it. I can't imagine one day if I cut them short." The ad ends with the tagline "some people don't know when to stop, it's the same with drinking".
Piercings	When to stop	Singapore	Government Health Promotion Board	60	A young woman with multiple face piercings explains "I got my first face piercing when I was 18. I've continued to grow the liking for it" as we see her lip being pierced. "Yeah as long as it makes me happy, there is never too much or an end to it". The ad ends with the tagline "some people don't know when to stop, it's the same with drinking".
Children and alcohol don't mix		United Kingdom	Balance	30	A child on one side of a room mimics his father on the other side, copying his clothing, movements and facial expressions. The voiceover says "guess what, kids copy, that's how they learn. If we show a responsible attitude to alcohol, so will they. 4 out of 5 people in the north east agree adults shouldn't drink too much in front of children."
Families and alcohol		United Kingdom	Balance	30	A young child reads a poem "my dad is always loving and kind, he is simply one of the best you'll find. A shiny example of everything good, my dad really loves me like a true father should" but the scenes show the father drunk, being a poor role model and missing his son's birthday. The end tagline is "is drinking affecting the ones you love?".
See what Sam sees		United Kingdom	Balance	45	In his daily life of catching the bus, in the supermarket, on the street, on TV, Sam takes note of all the alcohol ads and images he sees. By the time he is 12 years old he will have seen thousands of images. Alcohol ads encourage kids to start drinking younger and drink more. Join Balance to say enough is enough and sign the online petition.
The real price of cheap alcohol		United Kingdom	Balance	60	The ad starts with the questions: what price are we really paying for cheap alcohol? Our kids are paying the price: they are more like to say yes to illegal drugs, end up in hospital, a victim of violent crime, have unsafe sex. Alcohol harm costs the northeast tax payer £887 a year. Show your support for minimum unit price and sign the petition today.
Think twice		United Kingdom	Balance	60	Two glasses of wine are poured. "Many of us like to have a drink. We might pour a glass of wine like these, but did you know each of these glasses is half a pint of wine. It's important to know as there are proven links between alcohol and breast cancer. The next time you are having a drink, think twice about alcohol and breast cancer."
Tumour		United Kingdom	Balance	40	A man drinks beer as he is cooking. A tumour in the bottom of the glass grows as he drinks, and he swallows it with the last mouthful. "The World Health Organization classifies alcohol as a Group 1 Carcinogen; like tobacco and asbestos it can cause cancer. The more your drink and the more often you drink the more you increase your risk of cancer."
Bill Bailey	Alcohol. It's no joke	United Kingdom	Department for Children, Schools and Families	60	Bill Bailey performs a comedy show. He ends with a story about his neighbour's kid who went out drinking and he gets so smashed he can't remember where he lives. He staggers about, eventually recognising a familiar street corner when it hits him...the car. The lights slowly come on around Bill and we see he is standing beside an ambulance. "Alcohol. It's no joke".
Josie Long	Alcohol. It's no joke	United Kingdom	Department for Children, Schools and Families	60	Josie Long performs a comedy show. She ends with a story about her friend's stepsister who is 14 who recently went to her first ever house party, and it was the first time she was properly raging drunk and kissed a boy and 2 months later she was at the clinic for the first time. The lights slowly come on around Josie and we see her standing beside a hospital bed. "Alcohol. It's no joke".
Russell Kane	Alcohol. It's no joke	United Kingdom	Department for Children, Schools and Families	60	Russell Kane performs a comedy show. He ends with a story about a kid playing a game where you take a shot and then punch your mate and then it is their turn. It got really out of hand and this kid had just been punched, drank too much, and he smashed a glass and drove it into his best mate's face. The lights slowly come on around Russell and he is standing inside a jail cell. "Alcohol. It's no joke".

Change4Life		United Kingdom	Government Department of Health	60	This animated ad depicts a couple who enjoy having a few drinks to unwind at the end of the day. After depicting a number of health harms associated with alcohol (stroke, heart disease, mouth cancer etc.) they offer tips to reduce consumption, such as having a few alcohol free nights and swapping the hard stuff for something softer.
Binge boy	Know your limits	United Kingdom	Government Home Office	40	A young man gets ready for a night out by smashing his watch, ripping and spilling food down his t-shirt, urinating on his shoes, shaving off an eyebrow and ripping out an ear piercing. He looks in the mirror before walking out the door and the tagline states "you wouldn't start a night like this, so why end it that way?".
Binge girl	Know your limits	United Kingdom	Government Home Office	40	A young woman prepares for a night out by ripping her stockings and blouse, vomiting in the sink, smudging her makeup, spilling alcohol on her clothes and ripping the heel off her shoe. She checks herself in the mirror before walking out the door. The ad ends with the tagline "you wouldn't start a night like this so why end it that way?".
Catwalk	Know your limits	United Kingdom	Government Home Office	60	On a catwalk titled 'The Nightlife Collection', we see a young woman strutting the catwalk before squatting down and urinating. The next woman comes out and vomits on the catwalk. In the final scene, two male models get into a fight. The ad concludes with the tagline "you wouldn't start a night like this, so why end it that way?"
Street dares	Know your limits	United Kingdom	Government Home Office	80	In a shopping mall during the day, a young male films people's disgusted, scared and confused reactions to him acting drunk and trying to get them to join in singing offensive songs, imitating sexual acts, pouring vomit on their head, urinating in public, trying to start a fight, breaking a shop window. The ad ends with "If you wouldn't do it sober...".
Superhero	Know your limits	United Kingdom	Government Home Office	40	A bride-to-be loses some balloons that get caught high up on the scaffolding on a building. A superhero acrobatically climbs the scaffolding. The superhero reaches out for the balloons as the crowd watch on nervously, and then we see that the superhero is actually a man in a business suit. He then slips and falls. "Too much alcohol makes you feel invincible when you are most vulnerable".
Bloody Mary	Cocktales	United Kingdom	National Health Service	70	A group of young men walk along a busy street, drinking and talking to the women that they pass. Then they come across a young woman with smeared makeup who is urinating on the street. The men think it is a laugh until she falls over and starts bleeding. The ad ends with "too much alcohol ever ruined your night? Cocktales, get your mix right for the weekend."
Tequila slammer	Cocktales	United Kingdom	National Health Service	60	A group of young people in dress-up costumes are drinking and play fighting on a street footpath, when one of them is accidentally pushed in front of a passing car. There is a lot of screaming and someone calls for an ambulance. The ad ends with the tagline "too much alcohol ever ruined your night? Cocktales, get your mix right for the weekend."
Female	Damage you can't see	United Kingdom	National Health Service	40	Two women share a bottle of wine in the kitchen and one appears transparent as if by x-ray. The voiceover informs that alcohol increases the risk of high blood pressure, stroke and mouth cancer. The ad finishes with the women agreeing to finishing off the bottle, "well it can't do any harm". The ad ends with "drinking causes damage you can't see".
Male	Damage you can't see	United Kingdom	National Health Service	40	Three men are drinking in a pub and one appears transparent as if by x-ray. A voiceover informs that alcohol increases the risk of high blood pressure, stroke and mouth cancer. The ad finishes with the main character agreeing to another drink stating "one more can't hurt". The ad ends with the tagline "drinking causes damage you can't see".
Female units	Know your limits	United Kingdom	National Health Service	30	The ad depicts the number of units of alcohol the female character consumes over the course of a week (catching up with friends, eating at a restaurant, at home at the end of the week). The voiceover informs "if women regularly exceed 2-3 units a day, it can add up to a serious health problem".
Male units	Know your limits	United Kingdom	National Health Service	30	The ad depicts the number of units of alcohol the male character consumes over the course of a week (at the pub, eating at a restaurant, at home to unwind at the end of the week). The voiceover informs "if men regularly exceed 3-4 units a day it can add up to a serious health problem".
Don't be the one to miss out		United Kingdom	National Health Service and Brownlow Health	50	The ad opens with the question 'is this you?' while a young man is shown drinking at a pub. He pushes away water when offered and refuses to eat a meal with friends. Later on, he is vomiting outside. His friends put him to bed and laugh outside his bedroom door, and the text on screen reads 'don't be the one to miss out'.
Another night wasted		United Kingdom	National Health Service Northamptonshire	30	Sam had 5 wines and 2 double shots and is on the filthy floor beside the toilet with vomit all over her, she receives a text message that reads "where r u?". Joe had 5 pints of lager and 3 vodkas and is lying face down in pile of vomit in a toilet cubicle, a friend calls out that he is missing the soccer game. The ad ends with the tagline "another night wasted".
Binge Drinking. Your Night. Your Choice.		United Kingdom	Norfolk Constabulary	90	In one scenario a boy and girl meet at a pub, have a drink, dance and go home together. In the alternative, they drink excessively with their own group of friends separately, the girl doesn't feel well and leaves by herself, later we see her covered in vomit with a bloody nose. We also see the boy alone. The ad ends with police tape and "Binge drinking. Your night. Your choice".
Don't leave your brain at home		United Kingdom	Plymouth Community Safety Partnership and Devon Cornwall Police	30	This animation shows a brain left at home while a young man is out drinking, kissing girls, getting into a fight, and then arrested when someone spills his drink. The ad ends with the tagline "when you go drinking don't leave your brain at home".
Boy	You, your child and alcohol	United Kingdom	Police Service Northern Ireland and Public Health Agency	30	A father asks his teenage son about his night. He says he was at the park playing football. However, in the subtitles we see he was really drinking in the park, and that during a fight his friend was stabbed and so now he's scared. The ad ends with the voiceover "look out for the guide called 'You, your child and alcohol' for the truth about underage drinking."
Girl	You, your child and alcohol	United Kingdom	Police Service Northern Ireland and Public Health Agency	30	A mother asks her daughter about last night and she replies she was at a friend's place and they got Chinese food. However in the subtitles we see that she was really drinking in the park and then had sex with a friend of a friend. The ad ends with "look out for the guide called 'You, your child and alcohol' for the truth about underage drinking."
Underage drinking can ruin your life		United States of America	American Forces Network	30	A teenage girl says "People say I'm the life of the party", "Inhibitions? Not with me around", "I can get you do things you never dreamed you'd do", "I'll get you kicked off the team, wreck a few friendships, even trash your grades, but hey, aren't I worth it?", "I'm alcohol. And if you're really smart, you'll walk away". The ad ends with the tagline "underage drinking can ruin your life".
Binge drinking PSA		United States of America	Centers for Disease Control	80	A young male at a house party plays a game of beer pong-drinking beer through a funnel-but we see the harms associated with binge drinking going in the funnel instead of beer: memory loss, unwanted pregnancy, STIs, cancer, excessive vomiting, alcohol poisoning, and as he passes out we see death. The ad ends with him being wheeled out of the house on a ambulance stretcher.
Stupid ideas		United States of America	City of Edina, Minnesota	40	Two teenagers are seeking a mother's approval for what they could do for the afternoon. She rejects their ideas to juggle knives, ride a skateboard tied to a car, or to play 'chicken' and run across a highway. Their final idea is to grab some beers and go to the basement, which the mom agrees to. The ad ends with "letting underage kids drink... another stupid idea".
Female	Control your other you	United States of America	Clemson University, South Carolina	30	A young woman is putting on makeup in a bathroom. But in the reflection in the mirror we see how the woman behaves when she is intoxicated, along with the text "you don't undress in public... humiliate your friends... vandalize the campus... sleep around... but she has". The ad ends with the tagline "control your drinking, control your other you."
Male	Control your other you	United States of America	Clemson University, South Carolina	30	A respectable looking man is washing his hands in bathroom. But in the reflection in the mirror we see how the man behaves when he is intoxicated, along with the text "you've never threatened your wife... relieved yourself in public... groped your daughter's roommate... resisted arrest... but he has". The ad ends with "control your drinking, control your other you."
Beer and cookies		United States of America	Endina Police Department, Minnesota	100	A group of teenagers act as children playing skipping games and hopscotch. They are offered cookies by a mother. When she brings them out she also offers a beer, the teenagers look unsure. We then see the mother handcuffed and getting into a police car accompanied by a voiceover "ignoring underage drinking is the same as providing the bottle, it's time to stop looking the other way".
Are you a binge drinker?		United States of America	Marshall County Anti-Drug Coalition, Alabama	30	The ad asks 'are you a binge drinker?' and different voiceovers accompanying photos say "no way, it's a girls night out and it's only once in a while", "binge? it's a little fun at picnics, tailgating, its not like we drink all the time" and "it's a holiday party". The ad ends with "binge drinking is defined as consuming 5 drinks in one sitting."
Brandon	Start talking	United States of America	Substance Abuse and Mental Health Services Administration	30	At an alcoholic anonymous meeting, a child named Brandon explains that "in nine years I'll be an alcoholic, I'll start drinking with older kids and whatever they'll do, I'll do". A voiceover says "kids who drink before age 15 are 5 times more likely to have alcohol problems as adults, so start talking before they start drinking".
Dad	Talk. They hear you	United States of America	Substance Abuse and Mental Health Services Administration	60	In a garden shed a dad talks to his son about underage drinking, "alcohol can lead you to say and do things you wish you hadn't" and "if any of your buddies ever pressure you to take a drink just tell them you promised your dad you wouldn't". The ad ends with "they really do hear you, so start the conversation even before they are teenagers".
Mom	Talk. They hear you	United States of America	Substance Abuse and Mental Health Services Administration	60	A mother is asked by her 10 year old daughter if she can go to a sleepover. She is concerned, and asks her daughter to promise "that if there is any drinking I want you do say 'no thanks, not my thing'. Your real friends won't care". The ad ends with the voiceover "they really do hear you, so start the conversation even before they are teenagers".
