# Supplementary material for: Alcohol harm reduction advertisements: a content analysis of topic, objective, emotional tone, execution and target audience
Source: BMC Public Health. 2017 Apr 11;17:312. doi: 10.1186/s12889-017-4218-7 (PMC5387386; doi:10.1186/s12889-017-4218-7)
Supplement: Additional file 2: — Appendix B. Alcohol harm reduction advertising classification codebook. This file contains the detailed codebook used to classify alcohol harm reduction ads. The codebook consists of the list of variables examined and their definitions, along with the standardised response options. (PDF 65 kb) [file 12889_2017_4218_MOESM2_ESM.pdf]

APPENDIX B.

ALCOHOL HARM REDUCTION AD CLASSIFICATION CODEBOOK:

Coding values in blue text

MESSAGE TOPIC

Dominant topic (*select one only*)

What is the key information communicated in the ad? If multiple topics are presented code for the most dominant message, that is the one with the greatest air-time. Think about the main body of the ad when considering this code and not just the end frame.

1. Short-term harms of drinking (depicts short-term harms of drinking e.g. injury, violence, unsafe/unwanted sexual experience, vomiting, social embarrassment etc.)
2. Long-term harms of drinking (depicts long-term harms of drinking e.g. cancer, heart disease etc.)
3. Underage drinking (e.g. dangers of underage drinking, such as brain development, supply of alcohol to minors and adult drinking behaviours influencing those underage)
4. How to change behaviour (e.g. the ad may advise on how to reduce risk from alcohol harms and /or encourage positive change, such as tips for reducing alcohol consumption or looking after friends. Do not code yes if the ad only includes links to websites or telephone numbers for further information)
5. Advocacy / Policy change (e.g. the ad may advocate for policy change or request support for policy change)

BEHAVIOURAL OBJECTIVE

Behavioural objective (*select one only*)

What is the main objective or goal of the ad? That is, what is the main behaviour the ad is trying to elicit? Think about the main body of the ad when considering this code and not just the end frame.

1. Reduce alcohol consumption / limit drinking
2. Behave responsibly and/or don’t get drunk when drinking alcohol
3. Look after friends and family when they are drinking
4. Talk to a friend or family member about their drinking
5. Limit drinking when around children and teenagers
6. Never supply alcohol to teenagers
7. Parents to talk to their children about alcohol
8. Promote policy change

EMOTION

Emotional tone (*select one only*)

What type of emotional response is the ad trying to elicit among its target audience?

1. Negative emotions (e.g. fear, anxiety, disgust, worry, shame, guilt, regret, sadness etc.)
2. Positive emotions (e.g. hope, inspiration, determination, empowerment etc.)
3. Negative and positive emotions
4. Neutral (i.e. no emotion)

EXECUTIONAL CHARACTERISTICS

Guidelines (*select one only*)

Does the main body of the ad (excluding end frames) include any mention of drinking guidelines or recommended drinking limits to reduce harms?

0. No
1. Yes

Style (*select one only*)

What is the main executional style used in the ad?

1. Dramatization (ad is like a mini drama scene, with actors depicting a short story)
2. Simulation / Animation (ad involves simulation, animations or models of people, alcohol, key concepts etc.)
3. Factual (ad presents factual, medical, or statistical information without the use of simulation/animation, personal testimonial, or depicted scene (e.g., interview with spokesperson))
4. Personal testimonial (ad depicts a personalised narrative, a person telling “their story”)

Graphic imagery (*select one only*)

Does the ad include an explicit graphic or negative visceral image (an image eliciting an unrelieved visceral “ugh!” response) portraying the consequences of drinking alcohol?

0. No
1. Yes

Portrayal of drinking (*select one only*)

Does the ad display implicit or explicit portrayals of drinking alcohol?

1. Implicit portrayal only (alcoholic beverages being bought, ordered, held, poured, lid taken off etc. - but not actively being drunk)
2. Explicit portrayal (people actively drinking alcoholic beverage/s)
3. No portrayal of alcohol or consumption

Target audience: age or role (*select one only*)

Is this ad designed to appeal to a specific age group or specific roles people may have such as parents or governments? Cues may include direct references (e.g. "As a parent you need to know…"; "Join the 61% of young Victorians who…"), age of people in the ad, or other stylistic features of the ad such as music.

1. Children and adolescents (under 18 year olds)
2. Specifically targets young adults (~18-30 years)
3. Specifically targets parents
4. General adult audience
5. Government

Target audience: gender specific message (*select one only*)

Is this ad explicitly targeted to a specific gender? As indicated by direct references (e.g. "Females should limit to…"; "If you are a man drinking 2 pints of lager……". (Does not include ads that show only one gender drinking or being affected by drinking, unless accompanied by a direct reference).

0. Not gender specific / unknown
1. Yes (specifically targets males or females)

Subject of depicted harms/consequences of drinking (*select one only*)

Is the ad depicting the consequences of drinking for the target audience or for others?

1. Personal consequences for self (the target audience of the ad)
2. Consequences for others (other than the target audience of the ad [e.g. the ad targets parents but depicts consequences for children])
3. Both personal consequences for self and others (e.g. the ad targets friends/family members of drinkers and shows the consequences to both the target audience and the drinkers themselves)

OTHER VARIABLES CODED FOR BUT NOT INCLUDED IN PAPER:

EXECUTIONAL CHARACTERISTICS

Age of the central character in the ad (*select one only*)

What is the approximate age of the most central character/s in the ad? Central character/s are defined as the character/s who appears in the foreground, speaks during the ad, have screen time alone and/or appears for more than half the ad.

1. Children (~0-12 years)
2. Adolescents (~13-17 years)
3. Young adults (~18-30 years)
4. Older adults (~30+ years – use this code if character/s age appears on the cusp)
5. Multiple central characters in different age groups
6. Not applicable (e.g. no characters/people in ad, no central characters identifiable in ad)

Gender of the central character in the ad (*select one only*)

What is the gender of the central character/s in the ad?

1. Male
2. Female
3. Both (if there is more than one central character of different genders)
4. Not applicable (i.e. no characters/people in ad, no central characters identifiable in ad)

Drinking setting (*select one only*)

In what location does the drinking occur, includes both explicit and implicit (alcoholic beverages are seen in the setting but are not actively consumed)?

At home – inside or outside the home (enjoying a meal, house parties, backyard barbeques etc.)

0. No
1. Yes

In public – bars, pubs, restaurants, public parks, on the street etc.

0. No
1. Yes

Second-half punch (*select one only*)

Does the ad include a shocking, startling, or very surprising ending that a first-time viewer could not have anticipated? A second-half punch must occur in the second half of the advertisement.

0. No
1. Yes

End Frames (*select one only*)

What is the purpose of the end frames used in the ad? The end frames are defined as the final screens of the ad and the accompanying voiceover when the screens are displayed.

Reiteration of the key message/s and/or campaign slogans

0. No
1. Yes

Call to action / behaviour (includes statements such as "let's think about how much we drink" and "show your support, sign up today"; as well as directions to phone numbers, websites, further info etc.)

0. No
1. Yes

Guideline message (includes drinking guidelines or recommended drinking limits to reduce harm)

0. No
1. Yes

Target audience: drinkers vs. non-drinkers (*select one only*)

Is the ad explicitly targeted to drinkers or non-drinkers? Cues may include references to drinkers ("excessive drinking leads to your chance of being abused, injured or assaulted"; "binge drinking. your night. your choice"), non-drinkers ("don't start drinking just because your friends do, you're underage, no alcohol is the safest choice), or the ad may target the audience irrespective of their personal drinking status ("buy your children alcohol and they could pay the price"; "however you do it, tell them to ease up on the drink").

1. Drinkers (i.e. those who drink alcoholic beverages)
2. Non-drinkers (i.e. those who don't currently drink alcoholic beverages)
3. Targets audience irrespective of their drinking status (i.e. may be parents, whole communities, friends of drinkers etc.)
